# Impact of a Complement Factor H Gene Variant on Renal Dysfunction, Cardiovascular Events, and Response to ACE Inhibitor Therapy in Type 2 Diabetes

**DOI:** 10.3389/fgene.2019.00681

**Published:** 2019-07-26

**Authors:** Elisabetta Valoti, Marina Noris, Annalisa Perna, Erica Rurali, Giulia Gherardi, Matteo Breno, Aneliya Parvanova Ilieva, Ilian Petrov Iliev, Antonio Bossi, Roberto Trevisan, Alessandro Roberto Dodesini, Silvia Ferrari, Nadia Stucchi, Ariela Benigni, Giuseppe Remuzzi, Piero Ruggenenti

**Affiliations:** ^1^Aldo e Cele Daccò Clinical Research Center for Rare Diseases, Istituto di Ricerche Farmacologiche Mario Negri—IRCCS, Ranica, Italy; ^2^Units of Diabetology of Treviglio Hospital, Treviglio, Italy; ^3^Unit of Diabetology, Azienda Socio-Sanitaria Territoriale Papa Giovanni XXIII, Bergamo, Italy; ^4^Unit of Nephrology, Azienda Socio-Sanitaria Territoriale Papa Giovanni XXIII, Bergamo, Italy; ^5^Department of Biomedical and Clinical Sciences, University of Milan, Milan, Italy

**Keywords:** diabetes, complement, factor H, ACE inhibitors, microalbuminuria, cardiovascular risk, diabetes complications

## Abstract

Complement activation has been increasingly implicated in the pathogenesis of type 2 diabetes and its chronic complications. It is unknown whether complement factor H (CFH) genetic variants, which have been previously associated with complement-mediated organ damage likely due to inefficient complement modulation, influence the risk of renal and cardiovascular events and response to therapy with angiotensin-converting enzyme inhibitors (ACEi) in type 2 diabetic patients. Here, we have analyzed the c.2808G>T, (p.Glu936Asp) CFH polymorphism, which tags the H3 CFH haplotype associated to low plasma factor H levels and predisposing to atypical hemolytic uremic syndrome, in 1,158 type 2 diabetics prospectively followed in the Bergamo nephrologic complications of type 2 diabetes randomized, controlled clinical trial (BENEDICT) that evaluated the effect of the ACEi trandolapril on new onset microalbuminuria. At multivariable Cox analysis, the p.Glu936Asp polymorphism (Asp/Asp homozygotes, recessive model) was associated with increased risk of microalbuminuria [adjusted hazard ratio (HR) 3.25 (95% CI 1.46–7.24), *P* = 0.0038] and cardiovascular events [adjusted HR 2.68 (95% CI 1.23–5.87), *P* = 0.013]. The p.Glu936Asp genotype significantly interacted with ACEi therapy in predicting microalbuminuria. ACEi therapy was not nephroprotective in Asp/Asp homozygotes [adjusted HR 1.54 (0.18–13.07), *P* = 0.691 vs. non-ACEi-treated Asp/Asp patients], whereas it significantly reduced microalbuminuria events in Glu/Asp or Glu/Glu patients [adjusted HR 0.38 (0.24–0.60), *P* < 0.0001 vs. non-ACEi-treated Glu/Asp or Glu/Glu patients]. Among ACEi-treated patients, the risk of developing cardiovascular events was higher in Asp/Asp homozygotes than in Glu/Asp or Glu/Glu patients [adjusted HR 3.26 (1.29–8.28), *P* = 0.013]. Our results indicate that type 2 diabetic patients Asp/Asp homozygotes in the p.Glu936Asp CFH polymorphism are at increased risk of microalbuminuria and cardiovascular complications and may be less likely to benefit from ACEi therapy. Further studies are required to confirm our findings.

## Introduction

Complement activation products have been implicated in renal and cardiovascular complications of type 2 diabetes by promoting endothelial dysfunction and inflammation (Onat et al., 2011; Ghosh et al., 2015; Merle et al., 2015). Deposits of C3 activation fragments and of the terminal complement complex C5b-9 are observed in glomeruli and arteries of diabetic rats (Fischetti et al., 2011). The close association of these deposits with proteinuria, mesangial expansion, vascular hypertrophy, extracellular matrix deposition, and increased expression of adhesion molecules and growth factors strongly suggests that complement activation is involved in the onset and progression of renal and vascular complications of experimental diabetes (Fujita et al., 2013). Consistently, glomerular functional and structural changes are blunted by treatment with complement inhibitors, and vascular changes are almost fully prevented in C6-deficient diabetic rats that cannot form C5b-9 (Fujita et al., 1999; Fischetti et al., 2011).

In diabetic patients, elevated glomerular and tubular expression of C3 and Factor B—the two components of the alternative pathway C3 convertase C3bBb that cleaves C3 to C3a and C3b—has been associated with overt diabetic nephropathy (Woroniecka et al., 2011). In addition, plasma levels of Bb and C3a were higher in diabetic patients with nephropathy than in those without renal involvement (Li et al., 2018). Finally, plasma levels of C3 activation products correlated with the risk of severe atherosclerosis and ischemic heart disease in type 2 diabetics (Figueredo et al., 1993; Woroniecka et al., 2011; Fujita et al., 2013; Hertle et al., 2014; Li et al., 2018). The previously discussed findings converge to indicate that complement activation via the alternative pathway may contribute to the onset and progression of renal and vascular complications in human diabetes. This possibility is confirmed by the recent observation that among patients with diabetic nephropathy, the presence of deposits of C3 activation products in kidney biopsy was associated with lower renal function and more severe tubular and glomerular damage (Sun et al., 2018).

Should complement activation play a key role in the pathophysiology of chronic diabetic complications, then the availability of modulators of complement activity for clinical use would have major implications. Approximately one third of type 2 diabetics continue to develop micro- and macro-vascular disease, despite optimized metabolic and blood pressure control and early treatment with renin–angiotensin system (RAS) inhibitors. In these patients, renal and vascular dysfunctions, often heralded by a transition from normo- to microalbuminuria, substantially increase the risk of major renal and cardiovascular events, including overt nephropathy and progression to end-stage kidney disease, and coronary artery disease, myocardial infarction, and stroke (Holtkamp et al., 2011; Ruggenenti et al., 2011).

Complement factor H (CFH) plays a central role in the modulation of the complement alternative pathway by facilitating C3b degradation by the plasma serine protease factor I and enhancing C3 convertase dissociation (Jozsi and Zipfel, 2008). Factor H acts both in the fluid phase and on the endothelial cell surface, where it binds to heparan sulfate molecules and to C3 activation products deposited on cell membranes (Heinen et al., 2007). This is instrumental for protecting the host from excess complement activation and complement-mediated renal and vascular injury upon cell/tissue exposure to agents that may activate the alternative pathway. Consistently, reduced CFH bioavailability or activity, due to gene mutations or autoantibodies, may result in uncontrolled complement activation and consequent severe vascular damage, as observed in patients with atypical hemolytic uremic syndrome (aHUS), a rare thrombotic microangiopathy that targets the microvasculature of the kidney and other organs (Noris and Remuzzi, 2009). In addition, the CFH H3 common haplotype that includes polymorphisms in the promoter (rs3753394, c-331C>T) and the coding region of CFH (rs3753396, c.A2016G, p.Q672Q; and rs 1065489, c.2808G>T, p.Glu936Asp) confers an increased risk of aHUS and favors a poorer renal prognosis (Bernabeu-Herrero et al., 2015). Notably, among healthy controls, subjects homozygous for the H3 haplotype were found to have lower plasma CFH levels compared with individuals with zero H3 copies (Bernabeu-Herrero et al., 2015; Pouw et al., 2018). Furthermore, the previously mentioned CFH polymorphisms have been associated with susceptibility to other kidney diseases (Bonomo et al., 2014), eye diseases (Miki et al., 2014; Garcia et al., 2015; Wang et al., 2016), and infections (Davila et al., 2010; Zhang et al., 2013).

We postulated that activation of the complement system, possibly mediated by genetically determined reduced CFH bioavailability, could play a central role in the onset and progression of renal and vascular complications of diabetes. To explore whether and to what extent the CFH H3 haplotype may affect the risk of renal involvement in diabetes, we performed a post hoc analysis of the c.2808G>T (p.Glu936Asp) polymorphism, which is strongly associated with the H3 haplotype and determines an amino-acidic change close to the cysteine 931 involved in CFH folding (Reid and Day, 1989), in a large cohort of normoalbuminuric type 2 diabetics who were prospectively monitored through serial measurements of urinary albumin excretion (UAE) in the context of the Bergamo nephrologic complications of type 2 diabetes randomized, controlled clinical trial (BENEDICT) (Ruggenenti et al., 2004). Since the trial found that the ACE inhibitor (ACEi) trandolapril—alone or in combination with verapamil—reduced the risk of progression to microalbuminuria and the number of cardiovascular events (Ruggenenti et al., 2004; Ruggenenti et al., 2012), here we sought to explore whether and to what extent the c.2808G>T (p.Glu936Asp) *CFH* polymorphism could affect the interactions of diabetes and ACE inhibition with the previously discussed outcomes.

## Research Design and Methods

This is a post hoc analysis of the BENEDICT trial, a multicenter, double-blind, placebo-controlled, randomized clinical trial, designed to assess whether the ACEi trandolapril and the non-dihydropyridine calcium-channel blocker verapamil, alone or in combination, would prevent microalbuminuria in 1,204 subjects with hypertension, type 2 diabetes, and normal UAE. Detailed information about the trial is provided elsewhere (The BENEDICT Group, 2003; Ruggenenti et al., 2004).

Whereas the effect of verapamil was similar to that of the placebo, either trandolapril alone or in combination with verapamil reduced to a similar extent the risk of progression to microalbuminuria (The BENEDICT Group, 2003; Ruggenenti et al., 2004), a specific effect possibly related to RAS inhibition, which was independent of blood pressure and metabolic control. Thus, for the purpose of this study, patients were pooled in two cohorts according to their original allocation to ACEi or non-ACEi therapy regardless of concomitant therapy with verapamil or placebo: the ACEi group included patients treated with trandolapril or trandolapril plus verapamil, and the non-ACEi group included patients treated with verapamil or with placebo. Gene-by-treatment interactions were tested according to ACEi therapy (yes or no).

### Objectives

To investigate whether the p.Glu936Asp CFH variant could affect the risk of microalbuminuria and the protective effect of ACEi against this event, we genotyped the c.2808G>T (rs1065489) single nucleotide polymorphism (SNP) in 1,158 of the 1,204 type 2 normoalbuminuric diabetic patients included in the BENEDICT phase A study who consented to genetic analyses. Patients were actively followed until June 2004, when results of the final analysis became available (Ruggenenti et al., 2004). One hundred forty-seven nondiabetic volunteers from the same geographical region (Lombardy) served as healthy controls.

*Primary*—We first compared the allele frequency of the p.Glu936Asp CFH polymorphism between diabetic patients who developed microalbuminuria and those who did not develop microalbuminuria during follow-up. Thereafter, we aimed to evaluate the association between the p.Glu936Asp genotype according to different models and progression from normo- to microalbuminuria. We then evaluated the interactive role of the p.Glu936Asp CFH polymorphism and ACEi therapy in predicting new-onset microalbuminuria.

*Secondary*—Then, we evaluated the role of the previously mentioned interaction in predicting first onset of one of the components of a composite endpoint of fatal (including sudden death) or nonfatal major cardiovascular events, including events related to coronary (acute myocardial infarction, unstable angina pectoris, or coronary revascularization by bypass grafting or percutaneous transluminal angioplasty), cerebrovascular (stroke, transient ischemic attack, pre-cerebral artery revascularization) or peripheral artery (amputation, revascularization) disease, and hospitalization because of congestive heart failure.

*Competing events*—Patients who developed microalbuminuria were not censored, and their follow-up was continued until study end in order to also capture cardiovascular events occurring after progression to microalbuminuria. In addition, the risk that major cardiovascular events precluded the occurrence of microalbuminuria can be considered negligible. Indeed, during the BENEDICT phase A core trial, only five participants died from cardiovascular events. Moreover, the majority of patients experiencing nonfatal cardiovascular events remained in the core trial and had their albuminuria evaluated after the event.

### Definitions

The new onset of microalbuminuria was defined as UAE ≥20 and <200 μg/min in at least two of three consecutive overnight urine collections at two consecutive visits 2 months apart in previously normoalbuminuric (UAE < 20 μg/min in at least two out of three consecutive overnight samples) subjects (The BENEDICT Group, 2003; Ruggenenti et al., 2004). All cardiovascular events were adjudicated by two cardiologists (Brigitte Kalsh and Piero Ruggenenti) blinded to treatment and genetic analysis.

### Genotyping

Genomic DNA was extracted from peripheral blood leukocytes by Nucleon BACC2 kit (Amersham). Genotyping for c.2808G>T SNP of *CFH* was performed with Sanger direct sequencing using a primer pair designed to amplify exon 19 of CFH (Reid and Day, 1989) and the 3730-XL sequencer (Applied Biosystems).

### Ethics and Data Handling

The study was approved by the local ethics committee, and all study participants provided written informed consent according to the Helsinki Declaration guidelines. Data were handled with respect for patient confidentiality and anonymity.

### Statistical Analyses

The outcomes of interest were time to microalbuminuria or to major cardiovascular events. For patients who did not reach the endpoint, we censored time at the last follow-up visit with available data for albuminuria and at the last follow-up visit for major cardiovascular events.

All time-to-event endpoints were analyzed using Cox proportional hazard regression models, and results were expressed as hazard ratio (HR) and 95% confidence interval (CI). The Kaplan–Meier method was used to plot the probability of achieving the endpoints, according to the c.2808G>T (p.Glu636Asp) polymorphism and the ACEi treatment. Multivariable models for the endpoints included c.2808G>T (p.Glu636Asp) genotype, ACEi treatment and all the baseline covariates that, at the univariable Cox analysis, significantly (*P* < 0.05) associated with the outcome without exceeding the limit of one independent variable included in the model for every at least 10 outcome events available for the analyses. The previously discussed variables were also tested in Cox models with genotype × ACEi treatment interaction terms. In the multivariable approach, blood glucose was not considered because of its high colinearity with glycosylated hemoglobin (HbA1c) level that is a better biomarker of metabolic control as compared with blood glucose.

The covariates excluded in the univariable approach were considered in sensitivity analyses by testing each of the previously mentioned covariates in the multivariable models with genotype × ACEi treatment interaction. The same approach was used in exploratory statistical analyses to consider the predictive value of mean systolic blood pressure (SBP), diastolic blood pressure (DBP), mean arterial pressure, and Hba1C in the previously mentioned multivariable model, calculated on the basis of mean values during follow-up instead of baseline values.

Tests of the proportional hazard assumption were based on Schoenfeld residuals. To test possible differences between the four groups derived from genotype × ACEi treatment interaction, we used linear mixed effect models for DBP, SBP, and HbA1c. Not normally distributed covariates were log transformed before analysis. Normality for continuous variables was assessed by means of the Shapiro–Wilk and Kolmogorov–Smirnov tests.

Baseline characteristics were presented as numbers and percentages, means and standard deviations (SD), or medians and interquartile ranges (IQR), as appropriate. Comparisons between groups were made using unpaired t-test, Wilcoxon rank sum test, Chi-squared test, or paired t-test, as appropriate. Comparison between groups of UAE changes from baseline to the final visit was carried out by analysis of covariance. All P values were two-sided. Bonferroni correction was applied for multiple testing. Analyses were carried out using SAS (version 9.2) and Stata (version 13).

## Results

The genotype distribution of the c.2808G>T (p.Glu936Asp) C*FH* SNP in the 1,158 type 2 diabetics from the BENEDICT trial was comparable with the distribution in 145 nondiabetic, healthy volunteers with similar ancestry and geographic origin and did not deviate from the Hardy–Weinberg equilibrium (**Table 1**).

**Table 1:** Genotypic distribution of *CFH* SNP rs1065489 (c.2808 G>T, p.Glu936Asp) in type 2 diabetic patients of BENEDICT study and in healthy subjects.

c.2808 G>T	n	G/G	G/T	T/T	Hardy–Weinberg equilibrium
T2D patients	1,158	801	321	36	*X* ^2^ = 0.174; *P* = 0.917
Healthy subjects	145	97	46	2	*X* ^2^ = 0.971; *P* = 0.615
	*X * ^2^ = 2.152; *P* = 0.341				

### Complement Factor H Single Nucleotide Polymorphism Variants According to Progression to Microalbuminuria

**Figure 1:**
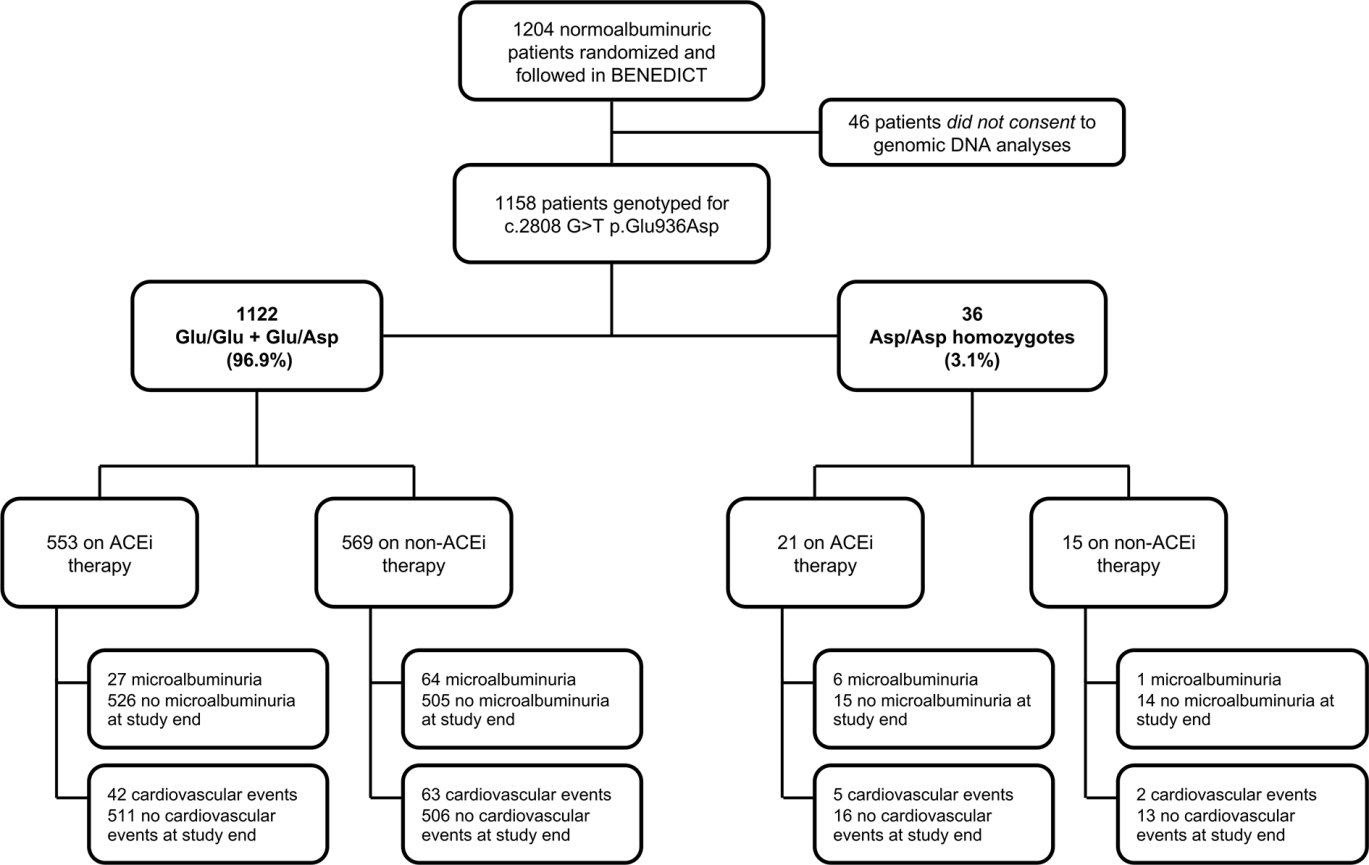
Study flow diagram of BENEDICT phase A type 2 diabetics screened for the p.Glu936Asp CFH SNP. ACEi: angiotensin-converting enzyme inhibitor.

Over a median (IQR) follow-up of 42 (12–51) months, 98 of the 1,158 participants (8.5%) progressed to new-onset microalbuminuria (**Figure 1**). Allele frequencies of the *CFH* c.2808G>T SNP differed significantly between patients with or without events (**Table 2**).

**Table 2:** Association analysis of genotypes and alleles of CFH c.2808G>T SNP with new-onset microalbuminuria in type 2 diabetic patients from BENEDICT phase A.

SNP	M/m	Renal events	Allele frequencies			Genotype frequencies				Additive model	Recessive model			Dominant model		
			M	m	*P*	MM	Mm	mm	P*	P*	MM/Mm	mm	P*	MM	Mm/mm	P*
rs1065489	G/T	Yes	0.77	0.23	**0.025**	0.612	0.316	0.072	*0.028*	*0.02*	0.928	0.072	*0.036*	0.612	0.388	0.096
c.2808G>T		No	0.836	0.164		0.699	0.274	0.027			0.973	0.027		0.699	0.301	

*A Bonferroni-corrected threshold of P = 0.0125 was considered as significant, while 0.0125 < P < 0.05 was considered suggestive of evidence for a potential association (in italics). P values in bold are statistically significant.

**Figure 2:**
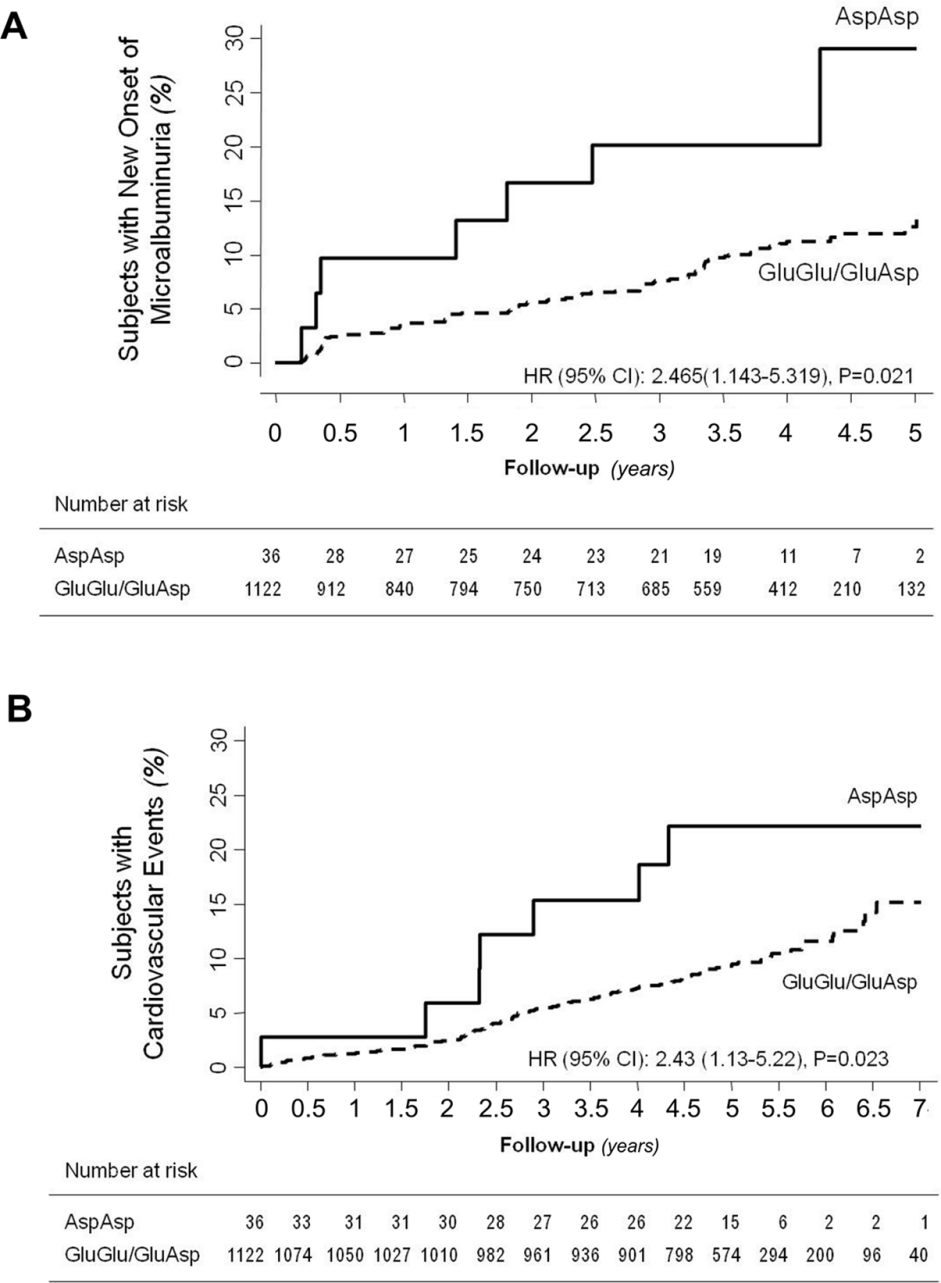
Impact of p.Glu936Asp CFH polymorphism on new-onset microalbuminuria and cardiovascular events. Kaplan–Meier curves show the fraction of Asp/Asp homozygotes or Glu/Glu+Glu/Asp diabetics who progressed to microalbuminuria (panel **A**) or developed cardiovascular events (panel **B**) throughout the study period. *P* values and HR (95% CI) of unadjusted Cox analyses are shown.

We found a suggestive evidence for potential association of the minor T variant (p.936Asp) with microalbuminuria, according to genotype distribution and the additive and the recessive models (**Table 2**). Considering the recessive model, 7 out of 36 Asp/Asp (T/T) homozygotes (19.4%) vs. 91 out of 1,122 Glu/Glu+Glu/Asp (TG/GG) patients (8.1%) developed new-onset microalbuminuria [Cox univariable analysis: HR (95% CI): 2.46 (1.14–5.32), *P* = 0.021, **Table 3**, Kaplan–Meier curve is shown in **Figure 2A**]. Cox univariable analysis with the additive model was not significant after Bonferroni correction [HR (95% CI): 1.4 (1.02–1.98), *P* = 0.04, **Table 3**, Kaplan–Meier curve is shown in **Supplementary Figure 1A**].

**Table 3:** Univariable Cox analyses for microalbuminuria and cardiovascular events.

	New-onset microalbuminuria			Cardiovascular events		
	Hazard ratio	95% CI	*P* value	Hazard ratio	95% CI	*P* value
p.Glu936Asp (*Rec model *)°	2.465	1.143–5.319	**0.0214**	2.425	1.128–5.215	**0.0233**
p.Glu936Asp (*Addit model *)°	1.417	1.016–1.977	*0.0401*	1.227	0.882–1.706	0.2251
Age (years)	1.014	0.989–1.040	0.2801	1.052	1.027–1.078	**<0.0001**
Gender (male)	2.339	1.494–3.663	**0.0002**	1.571	1.067–2.312	**0.0221**
BMI	1.023	0.983–1.064	0.2699	0.944	0.903–0.986	**0.0097**
Diabetes duration (years)	1.007	0.977–1.038	0.6393	1.013	0.985–1.041	0.3726
Hypertension duration (years)	0.996	0.967–1.027	0.8180	1.024	1.001–1.048	**0.0451**
Smoking habit	2.045	1.367–3.061	**0.0005**	1.139	0.785–1.652	0.4935
HbA1c*	6.788	2.936–15.696	**<0.0001**	2.828	1.271–6.293	**0.0108**
Glucose (mg/dl)	1.006	1.002–1.010	**0.0013**	1.004	1.000–1.007	**0.0488**
SBP (mmHg)	1.001	0.987–1.015	0.9004	1.000	0.987–1.013	0.9568
DBP (mmHg)	0.998	0.962–1.015	0.3873	0.979	0.955–1.004	0.0965
MAP (mmHg)	0.994	0.971–1.019	0.994	0.988	0.966–1.011	0.3135
Serum creatinine (mg/dl)	1.736	0.500–6.023	0.3851	4.286	1.390–13.214	**0.0113**
Triglycerides (mg/dl)*	0.978	0.654–1.463	0.9147	1.186	0.815–1.726	0.3740
Total cholesterol (mg/dl)	0.996	0.990–1.002	0.1530	1.004	0.999–1.009	0.1580
HDL cholesterol (mg/dl)*	0.865	0.406–1.845	0.7073	0.984	0.967–1.000	0.0558
LDL cholesterol (mg/dl)	0.995	0.989–1.001	0.0988	1.005	1.000–1.010	**0.0363**
UAE (μg/min)*	9.870	6.451–15.101	**<0.0001**	1.879	1.396–2.529	**<0.0001**
ACEi therapy	0.461	0.303–0.702	**0.0003**	0.722	0.496–1.050	0.0884
New onset micro	–	–	–	1.854	1.106–3.106	**0.0191**

°The Bonferroni-corrected threshold was P = 0.025. In bold are shown P value significant after Bonferroni correction; P values between 0.025 and 0.05 are shown in italics.

*Log transformed.

Rec, recessive model; Addit, additive model; MAP, mean arterial pressure; LDL, low-density lipoprotein; BMI, body mass index.

Baseline characteristics, the proportion of patients allocated to ACEi or non-ACEi therapy (**Table 4**), and the distribution of concomitant medications (**Supplementary Table 1**) were similar between the Asp/Asp and Glu/Glu+Glu/Asp genotype groups with the exception of a higher baseline HbA1c in Asp/Asp homozygotes [Asp/Asp: median 5.8% (IQR: 5.2–7.4), Glu/Glu+Glu/Asp: median 5.6 (IQR: 4.8–6.5), *P* < 0.05, **Table 4**].

**Table 4:** Clinical characteristics at randomization of patients with type 2 diabetes of BENEDICT phase A according to p.Glu936Asp CFH polymorphism.

	Asp/Asp homozygotes	Glu/Glu+Glu/Asp
n	36	1,122
Age (years)	63.4 (9.2)	62.2 (8.1)
Sex (male)	22 (61.1%)	585 (52.1%)
BMI	28.3 (26.1–32.5)	28.4 (25.8–31.6)
Diabetes duration (years)	7 (3–11.5)	6 (3–11)
Hypertension duration (years)	0.5 (0–7)	2 (0–8)
Smokers (current/former)	20 (55.6%)	463 (41.3%)
HbA1c (%)	5.8 (5.2–7.4)	5.6 (4.8–6.5)*
Glucose (mg/dl)	162.5 (40.1)	161.4 (47.1)
SBP (mmHg)	149.0 (14.5)	150.9 (14.1)
DBP (mmHg)	85.9 (7.6)	87.6 (7.6)
MAP (mmHg)	106.9 (8.7)	108.7 (8.3)
Serum creatinine (mg/dl)	0.92 (0.17)	0.91 (0.16)
Triglycerides (mg/dl)	137 (92–202)	125 (92–179)
Total cholesterol (mg/dl)	209.2 (22.7)	209.7 (37.0)
HDL cholesterol (mg/dl)	43 (36–57)	45 (38–54)
LDL cholesterol (mg/dl)	163.6 (25.2)	162.8 (36.1)
UAE (μg/min)	5.0 (3.6–9.2)	5.3 (3.6–9.3)
ACEi at randomization (yes)	21 (58.3%)	553 (49.3%)

Continuous variables are expressed as mean and SD and compared using unpaired t test or expressed as median and IQR and compared using Mann–Whitney test. Categorical variables are expressed in percentage and compared using Chi-square test or Fisher’s exact test as appropriate.

*P < 0.05 vs Asp/Asp homozygotes.

HbA1c, glycosylated hemoglobin; SBP, systolic blood pressure; DBP, diastolic blood pressure; UAE, urinary albumin excretion; MAP, mean arterial pressure; LDL, low-density lipoprotein; BMI, body mass index.

### Interactions Between p.Glu936Asp Genotype, Microalbuminuria, and Response to Angiotensin-Converting Enzyme Inhibitor Treatment

At univariable Cox analyses, the following covariates, male gender, smoking, and higher baseline UAE, HbA1c, and blood glucose were significantly associated with an increased risk of microalbuminuria, and ACEi therapy was associated with protection against this event (**Table 3**). Baseline UAE and HbA1c_,_ the p.Glu936Asp genotype (recessive model), and ACEi therapy retained an independent association with new-onset microalbuminuria at multiregression analysis (**Table 5**), which included all the baseline covariates that significantly predicted the outcome in the univariable approach (**Table 3**). With the additive model, no significant association was found between the p.Glu936Asp genotype and new-onset microalbuminuria at multiregression analysis (**Supplementary Table 3**).

**Table 5:** Multivariable Cox analysis for microalbuminuria and cardiovascular endpoints.

	Microalbuminuria number of events = 98		Cardiovascular events Number of events = 112	
	Hazard ratio (95% CI)	*P* value	Hazard ratio (95% CI)	*P* value
**p.Glu936Asp (*Rec model*)**	3.254 (1.462–7.241)	**0.0038**	2.683 (1.227–5.868)	**0.0134**
**ACEi therapy**	0.415 (0.270–0.639)	**<0.0001**	0.716 (0.485–1.055)	0.0908
**Gender (male)**	1.532 (0.913–2.569)	0.1061	1.610 (0.982–2.638)	0.0589
**Smoking habits**	1.401 (0.881–2.226)	0.1541	–	–
**HbA1c^†^**	5.170 (2.207–12.113)	**0.0002**	2.892 (1.231–6.797)	**0.0149**
**UAE^†^**	9.415 (6.042–14.791)	**<0.0001**	1.742 (1.262–2.404)	**0.0007**
**Age**	–	–	1.047 (1.020–1.075)	**0.0006**
**Hypertension duration**	–	–	1.026 (1.003–1.050)	**0.0250**
**BMI**	–	–	0.940 (0.895–0.986)	**0.0117**
**Serum Creatinine**	–	–	2.220 (0.595–8.287)	0.2352
**LDL cholesterol**	–	–	1.007 (1.002–1.012)	**0.0112**

^†^log transformed. Rec, recessive model; LDL, low-density lipoprotein; BMI, body mass index. P values in bold are statistically significant.

Among Asp/Asp homozygotes, there were more participants with insulin monotherapy in the non-ACEi group compared with the ACEi group (**Supplementary Table 1**). Among Glu/Glu+Glu/Asp patients, there were more patients receiving diuretics and sympatholytic agents, in the non-ACEi group, compared with the ACEi group (**Supplementary Table 1**).

In a Cox model including the p.Glu936Asp genotype (recessive model), ACEi therapy, and their interaction, the p.Glu936Asp genotype significantly interacted with ACEi therapy in predicting microalbuminuria [HR = 0.096, 95% CI (0.011–0.837), *P* = 0.034, **Table 6**].

**Table 6:** Multivariable Cox analysis with genotype–ACEi treatment interaction for microalbuminuria and cardiovascular endpoints *(without other covariates, reference: ACEi yes)*.

	New onset microalbuminuria Number of events = 98		Cardiovascular events Number of events = 112	
	Hazard ratio (95% CI)	*P* value	Hazard ratio (95% CI)	*P* value
p.Glu936Asp°	6.210 (2.563–15.044)	**<0.0001**	3.655 (1.445–9.242))	**0.006**
ACEi therapy	2.584 (1.648–4.052)	**<0.0001**	1.489 (1.007–2.200)	**0.046**
p.Glu936Asp*ACEi therapy	0.096 (0.011–0.837)	**0.034**	0.384 (0.071–2.073)	0.266

°Recessive model. P values in bold are statistically significant.

**Figure 3:**
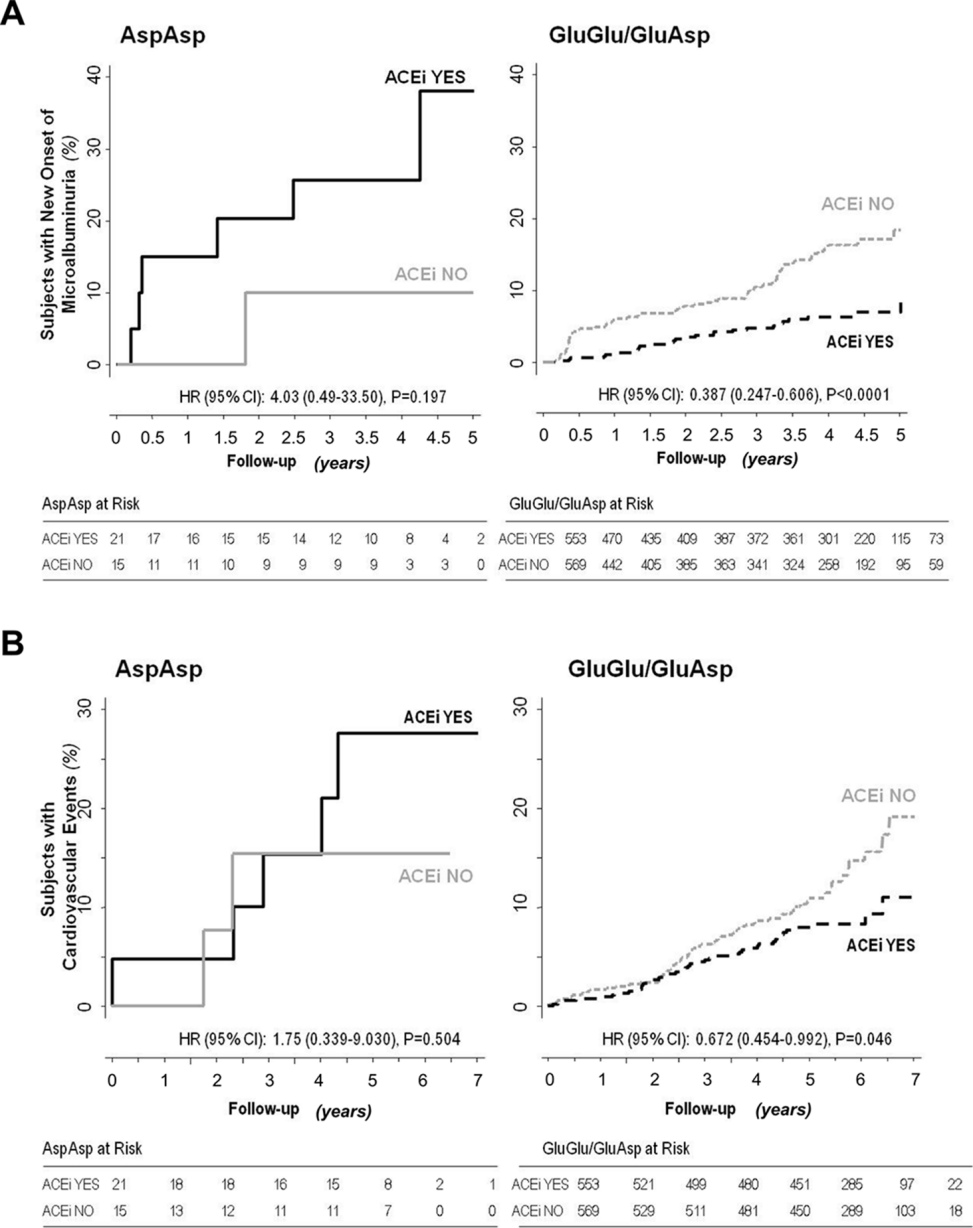
Impact of p.Glu936Asp CFH polymorphism and ACEi therapy on new-onset microalbuminuria and cardiovascular events. Kaplan–Meier curves show the fraction of Asp/Asp homozygous or Glu/Glu+Glu/Asp diabetic patients with or without ACEi therapy who progressed to microalbuminuria (panel **A**) or developed cardiovascular events (panel **B**) throughout the study period. P values and HR (95% CI) of unadjusted Cox analyses are shown.

**Figure 4:**
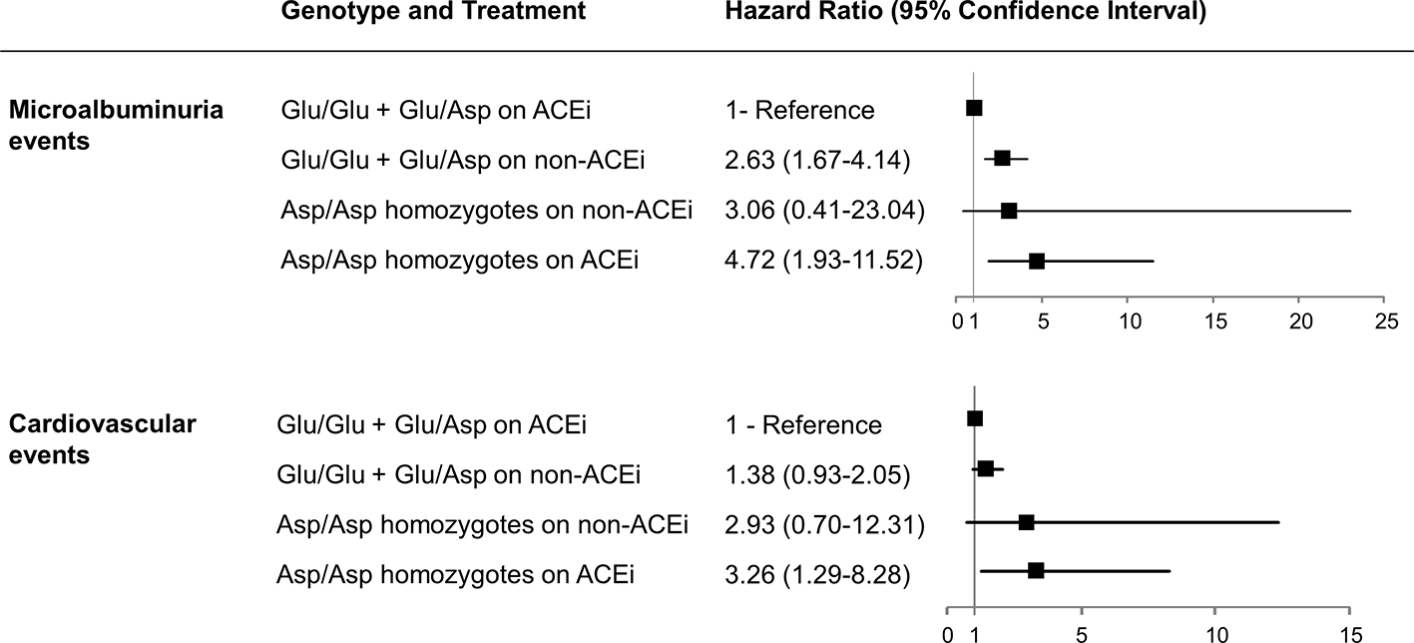
Hazard ratios for considered events according to p.Glu936Asp genotype and ACEi treatment. Adjusted hazard ratios (95% confidence intervals) for microalbuminuria and cardiovascular events according to p.Glu936Asp genotype and ACEi therapy compared with Glu/Glu+Glu/Asp patients on ACEi therapy taken as the reference group are shown.

Consistently, progression to microalbuminuria was observed in 6 of 21 Asp/Asp homozygotes on ACEi (28.6%) compared with 1 of 15 (6.7%) on non-ACEi [HR 4.03, 95% CI (0.49–33.50)] and in 27 of 553 (4.9%) Glu/Glu+Glu/Asp patients on ACEi compared with 64 of 569 (11.3%) on non-ACEi [HR 0.39 (0.25–0.61), *P* < 0.0001] (**Figure 1** and **Figure 3A**, **Supplementary Table 2**). Similar results were obtained after adjustment for baseline covariates that, at univariable analyses, were significantly associated with the event [**Table 7**, Asp/Asp homozygotes, ACEi vs. non-ACEi, adjusted HR 1.54, 95% CI (0.18–13.07), Glu/Glu+Glu/Asp ACEi vs. non-ACEi, adjusted HR 0.38, 95% CI (0.24–0.60) *P* < 0.0001]. Among ACEi-treated patients, Asp/Asp homozygotes had more microalbuminuria events than patients with Glu/Glu+Glu/Asp genotypes (adjusted HR 4.72, 95% CI [1.93–11.52], *P* = 0.001, **Table 7**), while among the non-ACEi patients, microalbuminuria events were comparable in the two genotype groups [adjusted HR 1.16, 95% CI (0.16–8.63), **Table 7**]. The adjusted HR for progression to microalbuminuria events increased progressively from 1 in Glu/Glu+Glu/Asp on ACEi (reference group) to 2.63 (95% CI 1.67–4.14, *P* < 0.0001) in Glu/Glu+Glu/Asp on non-ACEi, to 3.06 (95% CI 0.41–23.04) in Asp/Asp homozygotes on non-ACEi, and to 4.72 (95% CI 1.93–11.52, *P* = 0.001) in Asp/Asp on ACEi (**Table 7** and **Figure 4**).

**Table 7:** Panel A: HRs of the comparisons between ACEi-treated and non-ACEi-treated patients within the two genotype groups. Panel B: HRs of the comparisons between Asp/Asp homozygotes and Glu/Glu+Glu/Asp patients in ACEi or non-ACEi arms (genotype ACEi use interaction, with other covariates).

A	ACEi vs. non-ACEi	
	New-onset microalbuminuria	Cardiovascular events
Asp/Asp homozygotes	HR = 1.543, *P* = 0.691	HR = 1.112, *P* = 0.900
	95% CI (0.182–13.072)	95% CI (0.212–5.821)
Glu/Glu+Glu/Asp	HR = 0.381, ***P*** ** < 0.0001**	HR = 0.726, *P* = 0.113
	95% CI (0.241–0.601)	95% CI (0.488–1.079)
B	Asp/Asp homozygotes vs. Glu/Glu+Glu/Asp	
	New onset microalbuminuria	Cardiovascular events
non-ACEi	HR = 1.164, *P* = 0.882	HR = 2.128, *P* = 0.298
	95% CI (0.157–8.627)	95% CI (0.513–8.831)
ACEi	HR = 4.717, ***P*** ** = 0.001**	HR = 3.262, ***P*** ** = 0.013**
	95% CI (1.931–11.519)	95% CI (1.285–8.282)

P values in bold are statistically significant.

**Figure 5:**
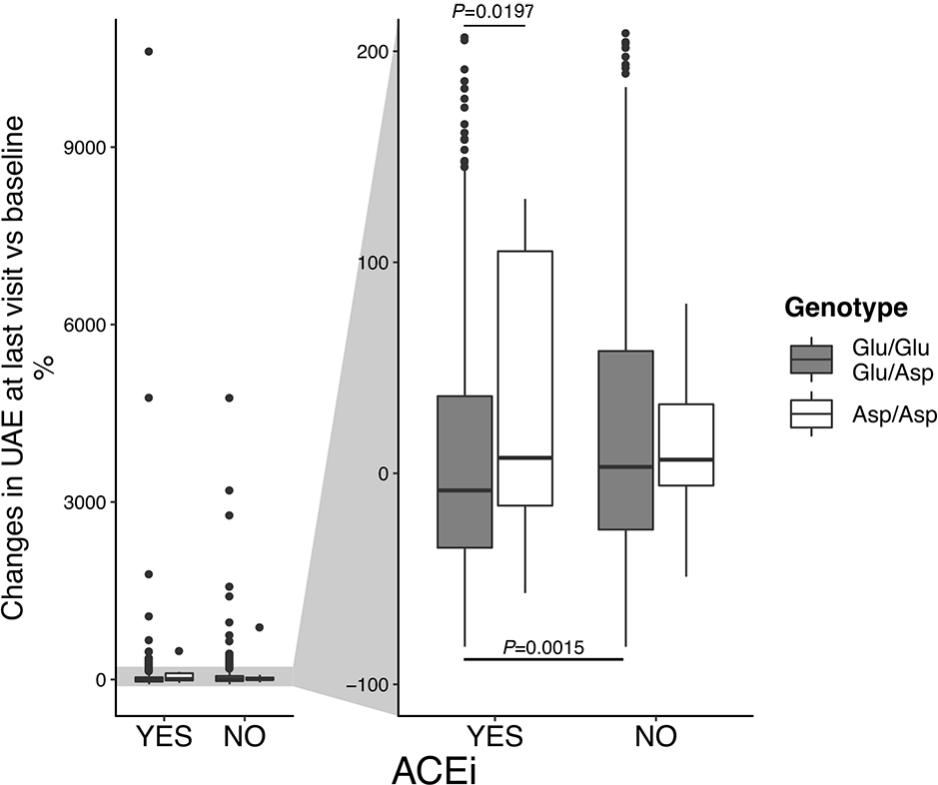
Changes in urinary albumin excretion (UAE) rate, according to p.Glu936Asp polymorphism and ACEi therapy. Percent changes of UAE at last visit vs. baseline. On the left, boxplots on the entire data range. On the right, boxplots were zoomed to show median (the central line) and IQR (lower and upper hinges).

Notably, among patients on non-ACEi, the risk of microalbuminuria events was comparable between Asp/Asp homozygotes and Glu/Glu+Glu/Asp patients (**Table 7**, **Figure 4**). Consistent findings were observed when the changes in albuminuria levels from baseline to the end of the study (or to new onset of microalbuminuria) were considered as a continuous variable (**Figure 5**). Thus, in ACEi-treated patients, UAE increased by 7% (median value) vs. baseline in Asp/Asp homozygotes, while it decreased by 8% in Glu/Glu+Glu/Asp patients. In non-ACEi patients, UAE increased by 6.5% and 3% in Asp/Asp homozygotes and in Glu/Glu+Glu/Asp patients, respectively (**Figure 5**). After adjustment for UAE at baseline, ACEi treatment did not impact on final UAE values in Asp/Asp homozygotes (*P* = 0.493 by analysis of covariance), whereas it significantly reduced UAE (*P* < 0.0015) in Glu/Glu+Glu/Asp patients compared with Glu/Glu+Glu/Asp on non-ACEi.

### Relationship Between Albuminuria and Cardiovascular Outcomes

Baseline UAE values were significantly associated with risk of cardiovascular events at both univariable (**Table 3**) and multivariable [HR 1.74 (95% CI 1.26–2.40), *P* = 0.0007] analyses (**Table 5**). There was also a significant association between new-onset microalbuminuria during the core study and the risk of cardiovascular events throughout the whole study period [**Table 3**, HR 1.85, 95% CI (1.11–3.11), *P* = 0.0191].

### Interactions Between p.Glu936Asp Genotype and Cardiovascular Events

During a median (IQR) follow-up of 60 (52–67) months, at least one major cardiovascular event was observed in 112 (9.6%) of the 1,158 patients (**Figure 1**). According to the recessive model, 7 of 36 Asp/Asp homozygotes (19.4%) vs. 105 of 1,122 Glu/Glu+Glu/Asp patients (9.4%) developed major cardiovascular events [Cox univariable analysis: HR 2.43, 95% CI (1.13–5.22), *P* = 0.023, **Table 3**, Kaplan–Meier curve is shown in **Figure 2B**]. At univariable and multivariable analyses, cardiovascular events were predicted by age, body mass index, hypertension duration, baseline UAE, HbA1c, and low-density lipoprotein cholesterol and by the p.Glu936Asp genotype (recessive model, **Tables 3** and **5**). With the additive model, no significant association was found between the p.Glu936Asp genotype and cardiovascular events either at univariable or multivariable analyses (**Table 3**, **Supplementary Figure 1B**, and **Supplementary Table 3**).

Among Asp/Asp homozygotes, major cardiovascular events were observed in 5 of 21 of those on ACEi (23.8%) and in 2 of 15 (13.3%) of those on non-ACEi [HR 1.75, 95% CI (0.34–9.03), **Supplementary Table 2** and **Figure 3B**], whereas among Glu/Glu+Glu/Asp patients, 42 of 553 (7.6%) of those on ACEi and 63 of 569 (11.1%) of those on non-ACEi [HR 0.67, 95% CI (0.46–0.99), *P* = 0.046, **Supplementary Table 2**, **Figure 1**, and **Figure 3B**] developed major cardiovascular events.

Adjustment for baseline covariates indicated that ACEi had no protective effect on cardiovascular risk in Asp/Asp patients [adjusted HR 1.11, 95% CI (0.21–5.82), **Table 7**], whereas it tended to reduce the risk of events [adjusted HR 0.73, 95% CI (0.49–1.08)] in the Glu/Glu+Glu/Asp group (**Table 7**), an effect that, however, failed to achieve statistical significance.

Among ACEi-treated participants, the risk of developing cardiovascular events was significantly higher in Asp/Asp homozygotes than in Glu/Glu+Glu/Asp subjects [**Table 7**, adjusted HR 3.26, 95% CI (1.29–8.28), *P* = 0.013], while in the non-ACEi group, no significant difference was found in the risk of cardiovascular events between Asp/Asp and Glu/Glu+Glu/Asp patients (**Table 7**).

The adjusted HR for developing an event increased progressively from Glu/Glu+Glu/Asp patients on ACEi (reference group), to Glu/Glu+Glu/Asp patients on non-ACEi [HR 1.38, 95% CI (0.93–2.05)], to Asp/Asp homozygotes on non-ACEi [HR 2.93, 95% CI (0.70–12.31)], and to Asp/Asp homozygotes on ACEi [HR 3.26, 95% CI (1.29–8.28), *P* = 0.013] (**Figure 4**).

### Adjustments for Blood Pressure and Metabolic Control

The relationships between the p.Glu936Asp genotype and microalbuminuria or cardiovascular events did not change appreciably when analyses were adjusted for SBP or DBP or HbA1C levels, either at baseline or mean levels during the study period (**Tables 8** and **9**).

**Table 8:** Multivariable Cox analysis for microalbuminuria based on 98 events.

	HbA1c fup + MAP fup		HbA1c fup + DBP fup		HbA1c fup + SBP fup	
	Hazard ratio (95% CI)	*P* value	Hazard ratio (95% CI)	*P* value	Hazard ratio (95% CI)	*P* value
p.Glu936Asp*	3.518 (1.568–7.893)	**0.0023**	3.702 (1.654–8.285)	**0.0014**	3.532 (1.572–7.932)	**0.0022**
**ACEi therapy**	0.483 (0.311–0.750)	**0.0012**	0.457 (0.295–0.707)	**0.0004**	0.491 (0.316–0.761)	**0.0015**
**Gender (male)**	1.584 (0.921–2.722)	0.0961	1.628 (0.943–2.811)	0.0804	1.689 (0.982–2.905)	0.0583
**Smoking habits**	1.439 (0.894–2.316)	0.1336	1.401 (0.872–2.250)	0.1635	1.433 (0.888–2.313)	0.0583
**UAE baseline^†^**	9.654 (6.085–15.315)	**<0.0001**	9.947 (6.291–15.729)	<0.0001	9.433 (5.922–15.027)	**<0.0001**
**HbA1c fup**	1.398 (1.192–1.639)	**<0.0001**	1.409 (1.204–1.649)	<0.0001	1.404 (1.197–1.648)	**<0.0001**
**MAP fup**	1.040 (1.004–1.077)	**0.0287**	– –	–	– –	–
**DBP fup**	– –	–	1.016 (0.976–1.058)	0.4365	– –	–
**SBP fup**	– –	–	– –	–	1.030 (1.010–1.050)	**0.0030**

fup, mean value during follow up; MAP, mean arterial pressure. *Recessive model. ^†^Log transformed. P values in bold are statistically significant.

**Table 9:** Multivariable Cox analysis for cardiovascular endpoints based on 112 CV events.

	Hba1c fup + MAP fup		Hba1c fup + DBP fup		Hba1c fup + SBP fup	
	Hazard ratio (95% CI)	*P* value	Hazard ratio (95% CI)	*P* value	Hazard ratio (95% CI)	*P* value
p.Glu936Asp*	2.406 (1.030–5.618)	**0.0425**	2.412 (1.036-5.618)	**0.0412**	2.441 (1.046-5.696)	**0.039**
**ACEi therapy**	0.887 (0.580–1.356)	0.5789	0.863 (0.564–1.320)	0.4973	0.853 (0.562–1.297)	0.458
**Gender (male)**	1.621 (0.932–2.819)	0.0869	1.637 (0.938–2.857)	0.0826	1.700 (0.983–2.943)	0.058
**HbA1c follow up**	1.090 (0.912–1.301)	0.3436	1.091 (0.914–1.303)	0.335	1.093 (0.916–1.304)	0.325
**UAE baseline^†^**	1.578 (1.109–2.247)	**0.0113**	1.601 (1.126–2.276)	**0.0087**	1.549 (1.086–2.208)	**0.0156**
**Age**	1.048 (1.109–1.078)	**0.0012**	1.055 (1.025–1.086)	**0.0002**	1.039 (1.008–1.070)	**0.0121**
**Hypertension duration**	1.007 (0.979–1.036)	0.6378	1.011 (0.0983–1.039)	0.454	1.007 (0.980–1.036)	0.615
**BMI**	0.924 (0.876–0.975)	**0.0039**	0.924 (0.875–0.975)	**0.004**	0.928 (0.880–0.978)	**0.0054**
**Serum creatinine**	1.760 (0.400–7.740)	0.454	1.680 (0.378–7.466)	0.4953	1.884 (0.431–8.234)	0.4
**LDL cholesterol**	1.008 (1.003–1.014)	**0.0032**	1.009 (1.003–1.014)	**0.0027**	1.009 (1.003–1.048)	**0.0026**
**MAP follow-up**	1.045 (1.010–1.081)	**0.0116**	– –	–	– –	–
**DBP follow-up**	– –	–	1.040 (1.002–1.081)	**0.0415**	– –	–
**SBP follow-up**	– –	–	– –	–	1.027 (1.005–1.048)	**0.0142**

MAP, mean arterial; BMI, body mass index; LDL, low-density lipoprotein. pressure. *recessive model. ^†^Log transformed. P values in bold are statistically significant.

## Discussion

In a large cohort of normoalbuminuric type 2 diabetics prospectively followed in the context of a randomized clinical trial (Ruggenenti et al., 2004), we found evidence indicating that carriers of the Asp/Asp genotype of the p.Glu936Asp CFH polymorphism were at a higher risk of progression to microalbuminuria than carriers of one or two wild-type Glu alleles. Surprisingly, the excess risk in Asp/Asp homozygotes tended to increase with ACEi therapy that exerted its expected renal protective effect only in Glu/Glu+Glu/Asp diabetic patients. Consequently, ACEi-treated Asp/Asp homozygotes were the patients at highest risk of new-onset microalbuminuria, whereas Glu/Glu+Glu/Asp patients on ACEi were those at the lowest risk. Consistently, ACEi therapy failed to prevent the progressive increase in albuminuria from baseline to the end of the study in Asp/Asp homozygotes, and its anti-albuminuric effect was restricted to carriers of one or two wild-type Glu alleles. These results may be taken to indicate that among normoalbuminuric type 2 diabetics, those with the CFH Asp/Asp genotype are at higher risk of renal involvement, and in this subset, the interaction between genotype, treatment, and outcome results in lack of response to the anti-proteinuric action of ACEi therapy.

Similar findings were observed when cardiovascular events were considered as outcomes. Again, the Asp/Asp genotype appeared to be associated with excess risk of cardiovascular events and poor responsiveness to ACEi. Thus, we find evidence indicating an incremental risk of cardiovascular events from ACEi-treated Glu/Glu+Glu/Asp diabetics to ACEi-treated Asp/Asp homozygotes, who were the patients at the highest risk of events.

Findings that risk of microalbuminuria and cardiovascular events, as well as the protective effect of ACEi against these events, were similarly affected by the underlying *CFH* genotype, are in harmony with consolidated evidence that renal and cardiovascular outcomes in subjects at risk are strongly associated (de Zeeuw et al., 2006). Consistently, both higher albuminuria at baseline and progression to microalbuminuria on follow-up significantly predicted cardiovascular events. Finding that outcome data did not appreciably change when analyses were adjusted for baseline and mean follow-up HbA1c and SBP and DBP values makes it possible to reasonably exclude major confounding effects of these concomitant risk factors.

In addition to modulating the alternative complement pathway in the fluid phase, CFH may bind specific sites on the renal microvascular endothelium and the glomerular capillary wall, to serve as a fixed complement regulator (Abrera-Abeleda et al., 2006). Thus, in experimental animals and humans, genetically determined CFH dysfunction results in uncontrolled alternative complement pathway activation, leading to complement-mediated renal endothelial damage (Pickering et al., 2007; Noris and Remuzzi, 2009). Consistently, the common *CFH*-H3 haplotype, including the T variant of the c.2808G>T (p.Glu936Asp) SNP, predisposes to aHUS, a rare disease characterized by complement-mediated glomerular endothelial injury (Caprioli et al., 2003; Pickering et al., 2007).

CFH is composed of 20 short consensus repeats (SCR) (Zipfel et al., 2006). The N-terminal SCRs 1–4 display complement regulatory activity, while the C-terminal SCRs 19–20 contain the recognition domain for cell surface proteoglycans and surface-bound C3 activation fragments (Zipfel et al., 2006). Once CFH C-terminus interacts with surface-bound C3b, CFH bends back on itself so that its N-terminus recognizes C3b and exerts its regulatory activity. The central SCRs 5–18, which connect N-terminal and C-terminal C3b-binding sites, are crucial for determining the flexibility required by CFH to achieve the folded bent-back structure and simultaneously occupy both sites on C3b (Morgan et al., 2011). Interestingly, the c.2808G>T SNP determines the glutammic (Glu) to aspartic (Asp) 936 amino-acidic change in SCR16 that is located 5 amino acids away from cysteine 931, which is crucial for S-S bridge formation and proper CFH folding (Reid and Day, 1989). Finding mutations affecting few amino acids away from the p.Glu936Asp polymorphism, in patients with genetically determined disease of the endothelium, (Bresin et al., 2013) provides additional evidence that a functional site located in SCR16 may be crucial to allowing CFH to acquire the suitable conformation to prevent complement activation on host cells. Moreover, the T variant of the c.2808G>T (p.Glu936Asp) SNP tags the CFH H3 haplotype, which was associated with lower CFH levels in subjects carrying two H3 copies as compared with subjects with zero copies (Bernabeu-Herrero et al., 2015; Pouw et al., 2018).

Altogether, the previously discussed observations would suggest that in Asp/Asp homozygous diabetic patients, conformational modifications and/or lower plasma concentration of CFH impair its protective effect on endothelial cells against the attack of complement components flowing in the blood, leading to endothelial dysfunction (Heinen et al., 2007). Endothelial dysfunction in renal microvasculature is a key factor in the early phase of diabetic nephropathy and may contribute to initial hyperfiltration and decreased permselectivity due to neo-angiogenesis and alterations in endothelial glycocalix (Nakagawa et al., 2011). It might also induce tubulo-interstitial injury in diabetes through plasma leaking from injured peritubular capillaries, with a secondary inflammatory tubulo-interstitial response (Temm and Dominguez, 2007). Complement activation products associated with diabetes (Ostergaard et al., 2005; Woroniecka et al., 2011; Fujita et al., 2013) could play a role in increased renal microvascular permeability, as suggested by the ability of C5a to cause endothelial cell retraction, gap formation, and increased fluorescein isothiocyanate–dextran passage through the cell monolayer (Schraufstatter et al., 2002; Khan et al., 2013). The circulating sC5b-9 complex also promotes vascular leakage of proteins and fluids (Bossi et al., 2004).

Thus, we suggest that the Asp936 CFH variant, in the context of the complement hyperactivation status associated with the diabetic milieu, regulates complement alternative pathway on the surface of endothelial cells less efficiently than the wild-type Glu936, which might result in renal endothelial dysfunction, albuminuria, and structural kidney damage.

Moreover, complement activation products might contribute directly to diabetic cardiovascular complications (Bjerre et al., 2008; Hertle et al., 2014) by promoting endothelial dysfunction and thrombotic events. In particular, C3a and C5a, and C5b-9, may induce endothelial cell detachment with exposure of subendothelium and secondary platelet aggregation and switch endothelial cells to a procoagulant phenotype due to expression of tissue factor (Tedesco et al., 1997; Schraufstatter et al., 2002). The excess of cardiac complications reported in patients with genetically determined endothelial disease associated with *CFH* mutations and chronic alternative pathway dysregulation (Noris et al., 2010) provides evidence that CFH dysfunction and consequent alternative pathway activation may play a major role in the pathogenesis of cardiovascular events in diabetics.

Why ACEi therapy had no protective effect against microalbuminuria or cardiovascular events in Asp/Asp homozygotes is a matter of speculation. It is well known that angiotensin II regulates the secretion of renin through a homeostatic mechanism-defined “feedback loop.” In animal models, the blockade of angiotensin II by ACEi causes an immediate and large increase in plasma renin concentration (Chen et al., 2010). A similar renin increase is documented in humans during treatment with ACEi (Johnston et al., 1979; Seifarth et al., 2002). Recently, Békássy and colleagues by *in vitro* studies provided evidence that renin triggers complement activation by cleaving C3 and generating functional C3b and C3a (Bekassy et al., 2018). Administration of a renin inhibitor in three patients with dense deposit disease (DDD), a rare kidney disease characterized by complement hyperactivation, decreased plasma C3a and C5a levels and complement deposition in the renal biopsy (Bekassy et al., 2018).

Based on these observations, we would speculate that in Asp/Asp homozygous diabetic patients treated with ACEi, an increase in renin plasma levels combined with defective CFH-mediated complement regulation could predispose to complement activation locally in the kidney, leading to the onset and progression of renal complications.

ACEi, by blocking the main plasma enzyme degrading bradykinin, may result in abnormally elevated levels of this potent vascular permeability factor, which occasionally associates with acute plasma extravasation and angioedema (Schmidt et al., 2010; Tomita et al., 2012). Finding that the permeabilizing activity of sC5b-9 *in vitro* and *in vivo* is inhibited by a bradykinin 2 receptor antagonist suggests a cross talk between the complement and the kinin system in inducing vascular permeability (Bossi et al., 2004).

A plausible explanation of our findings could be that in Asp/Asp diabetics, ineffective modulation of the complement alternative pathway combined with excess renin and bradykinin levels associated with ACEi treatment might enhance complement activation and microvascular permeability and offset the anti-proteinuric and cardioprotective effects of RAS blockade.

### Limitations and Strengths

This was a post hoc analysis of a study originally designed for other purposes. The number of cardiovascular events was relatively small, which limited the statistical power to evaluate possible interactions of baseline covariates and treatment with cardiovascular outcomes. Replication studies are needed to confirm the association between the p.Glu936Asp CFH polymorphism and the risk of renal and cardiovascular complications. This study was focused on the analysis of a single SNP selected on the basis of the hypothesis that the p.Glu936Asp CFH polymorphism that tags the H3 haplotype and has been found in association with a rare kidney disease with microvascular involvement may play a predisposing role in microvascular complications of diabetes. On the other hand, we recognize that a genome-wide analysis would address the multiple testing approach and would allow to assess population stratification, possible cryptic relatedness, and to evaluate pathways.

Major strengths were standardized study conduction, centralized measurement of all considered laboratory variables, including microalbuminuria—which was assessed by gold standard procedures in triplicate overnight urine collections (Ruggenenti et al., 2004)—and the centralized adjudication of cardiovascular events by two cardiologists who were blinded to treatment allocation. Study findings are widely generalizable because more than 96% of the BENEDICT population was genotyped and data were observed in type 2 diabetics with normoalbuminuria and hypertension, a typology of patients that accounts for at least 90% of the whole diabetic population.

Finally, at variance with most previous similar genetic studies that included also patients with micro- or macroalbuminuria who were largely driving the study findings, or did not measure albuminuria levels, our study investigated the impact on renal outcome of the p.Glu936Asp CFH polymorphism in a pure population of patients with no evidence of renal involvement at baseline (Parving et al., 2008; Bacci et al., 2011; Mooyaart et al., 2011; Ahlqvist et al., 2015).

### Conclusions

Screening for the p.Glu936Asp polymorphism may help identify subjects at increased risk of microalbuminuria and cardiovascular events and those who might not benefit from ACEi therapy. Notably, observational evidence that ACEi therapy was associated with an excess risk of events in patients with the Asp/Asp CFH genotype suggests that renal and cardiac effects of ACEi in diabetes may be influenced by genetic variations and highlights the urgent need for genome-wide studies to primarily address this critical issue in larger number of patients.

The present findings should not be taken as a reason for not offering ACEi therapy to type 2 diabetic patients who in general may benefit of nephro- and cardio-protective effects of this class of drugs. Instead, our data indicate that genotyping for the c.2808G>T (p.Glu936Asp) polymorphism could help identifying a subpopulation of patients, accounting for approximately 3% of type 2 diabetics, who are less likely to benefit from these drugs to avoid unnecessary exposure to potentially serious, treatment-related side effects.

## Data Availability

The raw data supporting the conclusions of this manuscript will be made available by the authors, without undue reservation, to any qualified researcher.

## Ethics Statement

The study was approved by the Ethics Committee of Azienda Sanitaria Locale, Bergamo, Italy. All study participants provided written informed consent according to the Helsinki Declaration guidelines. Data were handled with respect for patient confidentiality and anonymity.

## Author Contributions

EV, MN, GR, and PR designed research, interpreted data, and wrote the paper; EV and ER performed the research and analyzed the data; AP and MB performed statistical analyses; GG organized blood and informed consent collection; API, IPI, AB, RT, and AD participated to BENEDICT study and provided detailed clinical information of patients; SF and NS performed laboratory measurements; AB analyzed the data and critically revised the manuscript. GR was the principal investigator of BENEDICT study. PR was the study coordinator of the BENEDICT study.

## Funding

This study was partially supported by a grant from Fondazione ART per la Ricerca Sui Trapianti ONLUS (Milan, Italy). EV and MB are recipients of research contracts supported by Progetto DDD Onlus—Associazione per la lotta alla DDD (Milan, Italy) and Cassa di Sovvenzioni e Risparmio fra il Personale della Banca D’Italia (Rome, Italy). BENEDICT was supported by Abbott (Ludwigshafen, Germany). The funding sources had no role in study design and conduction and in paper finalization and submission.

## Conflict of Interest Statement

BENEDICT was supported by Abbott (Ludwigshafen, Germany). The funding sources had no role in study design and conduction, and in paper finalization and submission.

MN has received honoraria from Alexion Pharmaceuticals for giving lectures and participating in advisory boards. None of these activities have had any influence on the results or their interpretation in this article. GR has consultancy agreements with AbbVie*, Alexion Pharmaceuticals*, Bayer Healthcare*, Reata Pharmaceuticals*, Novartis Pharma*, AstraZeneca*, Otsuka Pharmaceutical Europe*, and Concert Pharmaceuticals*.

*No personal remuneration is accepted; compensation is given to his institution for research and educational activities.

The remaining authors declare that the research was conducted in the absence of any commercial or financial relationships that could be construed as a potential conflict of interest.
