# Supplementary material for: Impact of a Complement Factor H Gene Variant on Renal Dysfunction, Cardiovascular Events, and Response to ACE Inhibitor Therapy in Type 2 Diabetes
Source: Front Genet. 2019 Jul 26;10:681. doi: 10.3389/fgene.2019.00681 (PMC6689971; doi:10.3389/fgene.2019.00681)

#### Supplementary Materials

#### The Benedict Study Group Organization

Members of the BENEDICT Study Organization were as follows (all in Italy unless otherwise noted): Principal investigator — G. Remuzzi (Bergamo); Study coordinator — P. Ruggenenti (Bergamo); Coordinating center — IRCCS Mario Negri Institute for Pharmacological Research, Clinical Research Center for Rare Diseases Aldo e Cele Daccò, Villa Camozzi, Ranica (Bergamo); Participating centers — R. Trevisan, A.R. Dodesini, G.Lepore, I. Nosari, A. Fassi (Bergamo); A. Belviso, M. Trillini (Ponte San Pietro); P. Cravedi, C. Chiurchiu, S. Rota, S. Prandini (Ranica); A. Bossi, A. Parvanova, I.P. Iliev, V. Lecchi, S. Yakymchuk (Romano di Lombardia, Treviglio); R. Mangili, (Seriate); Ophthalmologists — M. Filipponi, I.P. Iliev, S. Tadini (Bergamo); Monitoring and Drug Distribution (Mario Negri Institute) — N. Rubis, G. Gherardi, W. Calini,O. Diadei, M. Lesti, D. Rossoni, D. Villa (Ranica); Carriers (Mario Negri Institute)—G. Gaspari, S. Gelmi, (Ranica);Database and Data Validation (Mario Negri Institute) — A. Remuzzi, B. Ene-Iordache, S. Carminati (Ranica); Data Analysis (Mario Negri Institute) — A. Perna, N. Motterlini, A. Chianca (Ranica); Laboratory Measurements (Mario Negri Institute) — F. Gaspari, F. Carrara, S.Ferrari, N. Stucchi, A. Cannata, M. Galbusera, S. Gastoldi, C. Tentori (Ranica, Bergamo); Genetic Analyses (Mario Negri Institute) — M. Noris, E. Valoti, E.Rurali, R. Donadelli, S. Nosari (Ranica); Organization of blood and informed consent collection (Mario Negri Institute) — G. Gherardi, D. Cugini (Ranica) Regulatory Affairs (Mario Negri Institute) — P. Boccardo (Ranica); Steering Committee — L. Minetti (Bergamo), G. Remuzzi (Bergamo), U.F. Legler (Ludwigshafen,Germany), B. Kalsch (Ludwigshafen, Germany), D. Nehrdich (Ludwigshafen,Germany), A. Nicolucci (S. Maria Imbaro), A. Perna (Bergamo), P. Ruggenenti (Bergamo); Safety Committee — G.L. Bakris (Chicago, United States), R. Kay (Sheffield, United Kingdom), G.C. Viberti (London, United Kingdom); Approvement of Bioethical Committee of the ASL in Bergamo (Italy).

#### Supplementary Tables

**Supplementary Table 1. Concomitant treatments in patients with type 2 diabetes at baseline and during follow-up.**

| **Treatment** | **Baseline**  *Number (percent)* | | | | | | **Follow-up**  *Number (percent)* | | | | | |
| --- | --- | --- | --- | --- | --- | --- | --- | --- | --- | --- | --- | --- |
|  | **Asp/Asp homozygotes** | | | **Glu/Glu + Glu/Asp** | | | **Asp/Asp homozygotes** | | | **Glu/Glu + Glu/Asp** | | |
| **Concomitant medication** | **Overall**  **(n = 36)** | **ACEi**  **(n = 21)** | **non-ACEi**  **(n = 15)** | **Overall**  **(n = 1122)** | **ACEi**  **(n = 553)** | **non-ACEi**  **(n = 569)** | **Overall**  **(n = 36)** | **ACEi**  **(n = 21)** | **non-ACEi**  **(n = 15)** | **Overall**  **(n = 1122)** | **ACEi**  **(n = 553)** | **non-ACEi**  **(n = 569)** |
| *Glucose-lowering regimen* |  |  |  |  |  |  |  |  |  |  |  |  |
| Diet alone | 11 (30.6) | 7 (33.3) | 4 (26.7) | 333 (29.7) | 162 (29.3) | 171 (30.1) | 8 (22.2) | 4 (19.1) | 4 (26.7) | 229 (20.4) | 114 (20.6) | 115 (20.2) |
| Oral hypoglycemic agent alone | 17 (47.2) | 10 (x47.6) | 7 (46.7) | 656 (58.5) | 331 (59.9) | 325 (57.1) | 18 (50.0) | 11 (52.4) | 7 (46.7) | 694 (61.9) | 344 (62.2) | 350 (61.5) |
| Insulin and oral hypoglycemic agent | 4 (11.1) | 4 (19.1) | 0 (0.0) | 72 (6.4) | 29 (5.2) | 43 (7.6) | 8 (22.2) | 6 (28.6) | 2 (13.3) | 140 (12.5) | 62 (11.2) | 78 (13.7) |
| Insulin alone | 4 (11.1) | 0 (0.0) | 4 (26.7)* | 61 (5.4) | 31 (5.6) | 30 (5.3) | 2 (5.6) | 0 (0.0) | 2 (13.3) | 59 (5.3) | 33 (6.0) | 26 (4.6) |
| *Antihypertensive agents* |  |  |  |  |  |  |  |  |  |  |  |  |
| Any | 18 (50.0) | 10 (46.7) | 8 (53.3) | 616 (54.9) | 303 (54.8) | 313 (55.0) | 24 (66.7) | 14 (66.7) | 10 (66.7) | 691 (61.6) | 325 (58.8) | 366 (64.3) |
| Diuretic | 13 (36.1) | 7 (33.3) | 6 (40.0) | 233 (20.8) | 107 (19.4) | 126 (22.1) | 9 (25.0) | 5 (23.8) | 4 (26.7) | 209 (18.6) | 89 (16.1) | 120 (21.1)* |
| Beta-blocker | 3 (8.3) | 1 (4.8) | 2 (13.3) | 91 (8.1) | 49 (8.9) | 42 (7.4) | 5 (13.9) | 3 (14.3) | 2 (13.3) | 93 (8.3) | 46 (8.3) | 47 (8.3) |
| Calcium-channel blocker (dihydropyridine) | 4 (11.1) | 3 (14.3) | 1 (6.7) | 322 (28.7) | 148 (26.8) | 174 (30.6) | 9 (25.0) | 5 (23.8) | 4 (26.7) | 311 (27.7) | 143 (25.9) | 168 (29.5) |
| Sympatholytic agent | 8 (22.2) | 5 (23.8) | 3 (2.0) | 232 (20.7) | 118 (21.3) | 114 (20.0) | 17 (47.2) | 9 (42.9) | 8 (53.3) | 521 (46.4) | 228 (41.2) | 293 (51.5)° |
| *Lipid-lowering agents* |  |  |  |  |  |  |  |  |  |  |  |  |
| Any | 4 (11.1) | 3 (14.3) | 1 (6.7) | 121 (10.8) | 61 (11.0) | 60 (10.5) | 5 (13.9) | 4 (19.1) | 1 (6.7) | 216 (19.3) | 99 (17.9) | 117 (20.6) |
| Statin alone | 3 (8.3) | 3 (14.3) | 0 (0.0) | 79 (7.0) | 44 (8.0) | 35 (6.2) | 4 (11.1) | 4 (18.1) | 0 (0.0) | 164 (14.6) | 76 (13.7) | 88 (15.5) |
| Fibrate alone | 0 (0.0) | 0 (0.0) | 0 (3.4) | 38 (2.5) | 14 (4.2) | 24 (0.0) | 0 (0.0) | 0 (0.0) | 0 (0.0) | 42 (3.7) | 18 (3.3) | 24 (4.2) |
| Statin and fibrate | 1 (2.8) | 0 (0.0) | 1 (6.7) | 3 (0.3) | 2 (0.4) | 1 (0.2) | 1 (2.8) | 0 (0) | 1 (6.7) | 20 (1.8) | 11 (2.0) | 9 (1.6) |
| *Antiplatelet agent* | 0 (0.0) | 0 (0.0) | 0 (0.0) | 27 (2.4) | 14 (2.5) | 13 (2.3) | 4 (11.1) | 3 (14.3) | 1 (6.7) | 86 (7.7) | 46 (8.3) | 40 (7.0) |

***P<0.05 vs ACEi, °P<0.001 vs ACEi.**

**Supplementary Table 2. Panel A: HRs of the comparisons between ACEi treated and non-ACEi treated patients within the two genotype. Panel B: HRs of the comparisons between Asp/Asp homozygotes and Glu/Glu + Glu/Asp patients in ACEi or non-ACEi arms *(genotype, ACEi use, interaction without other covariates).***

| **A** | **ACEi vs. non-ACEi** | |
| --- | --- | --- |
|  | **Microalbuminuria events** |  |
|  |  | **Cardiovascular events** |
| **Asp/Asp homozygotes** | HR=4.030, P=0.197 | HR=1.750 P=0.504 |
|  | 95%CI (0.485-33.496) | 95%CI [0.339-9.030] |
| **Glu/Glu +** | HR=0.387, **P<0.0001** | HR=0.672 **P=0.046** |
| **Glu/Asp** | 95%CI (0.247-0.607) | 95%CI [0.455-0.993] |
|  |  |  |
| **B** | **Asp/Asp homozygotes vs. Glu/Glu + Glu/Asp** | |
|  | **Microalbuminuria events** |  |
|  |  | **Cardiovascular events** |
| **non-ACEi** | HR=0.596, P=0.608 | HR=1.403, P=0.470 |
|  | 95%CI (0.083-4.300) | 95%CI (0.343-5.737) |
| **ACEi** | HR=6.210, **P<0.0001** | HR=3.655, **P=0.006** |
|  | 95%CI (2.563-15.043) | 95%CI (1.445-9.242) |

**Supplementary Table 3. Multivariable Cox analysis for microalbuminuria and cardiovascular end points with the p.Glu936Asp additive model.**

|  | | **Renal events**  **Number of events**=98 | | | | |  | **Cardiovascular events**  **Number of events**=112 | | | |
| --- | --- | --- | --- | --- | --- | --- | --- | --- | --- | --- | --- |
|  |  | Hazard Ratio | P value | | | |  | Hazard Ratio | | P value | |
|  |  | (95% CI) |  |  |  |  |  | (95% CI) | |  |  |
| **p.Glu936Asp**  ***(Addit model)*** | | 1.348 | 0.0932 | | | |  | 1.217 | | 0.2562 | |
|  |  | (0.951-1.912) |  |  |  |  |  | (0.867-1.708) | |  | |
| **ACEi therapy** | | 0.430 | **0.0001** | | | |  | 0.734 | | 0.1153 | |
|  |  | (0.281-0.660) |  | | | |  | (0.499-1.079) | |  | |
| **Gender (male)** | | 1.570 | 0.0878 | | | |  | 1.678 | | **0.0395** | |
|  |  | (0.935-2.636) |  | | | |  | (1.025-2.747) | |  | |
| **Smoking habits** | | 1.463 | 0.1040 | | | |  | - | | **-** | |
|  |  | (0.925-2.316) |  | | | |  |  | |  | |
| **HbA1c^†^** | | 5.434 | **<0.0001** | | | |  | 2.924 | | **0.0140** | |
|  |  | (2.318-12.738) |  | | | |  | (1.243-6.878) | |  | |
| **UAE^†^** | | 8.718 | **<0.0001** | | | |  | 1.715 | | **0.0010** | |
|  |  | (5.634-13.489) |  | | | |  | (1.243-2.365) | |  | |
| **Age** | | **-** | - | | | |  | 1.047 | | **0.0007** | |
|  |  |  |  | | | |  | (1.019-1.075) | |  | |
| **Hypertension duration** | | **-** | **-** | | | |  | 1.029 | | **0.0176** | |
|  |  |  |  | | | |  | (1.005-1.053) | |  | |
| **BMI** | | **-** | **-** | | | |  | 0.938 | | **0.0104** | |
|  |  |  |  | | | |  | (0.894-0.985) | |  | |
| **Serum Creatinine** | | **-** | **-** | | | |  | 2.012 | | 0.2951 | |
|  |  |  |  | | | |  | (0.544-7.444) | |  | |
| **LDL cholesterol** | **-** | | | **-** |  | **1.007** | | | **0.0121** | |  |
|  |  | | |  |  | **(1.001-1.012)** | | |  | |  |

^†^ log transformed.

Legend to supplementary figure 1

**Supplementary Figure 1. Impact of p.Glu936Asp CFH genotypes on new-onset microalbuminuria and cardiovascular events.** Kaplan-Meier curves show the fraction of Asp/Asp homozygous, Glu/Asp heterozygous or Glu/Glu homozygous diabetics who progressed to microalbuminuria (panel A) or developed cardiovascular events (panel B) throughout the study period. P values and HR (95% CI) of unadjusted Cox analyses are shown.


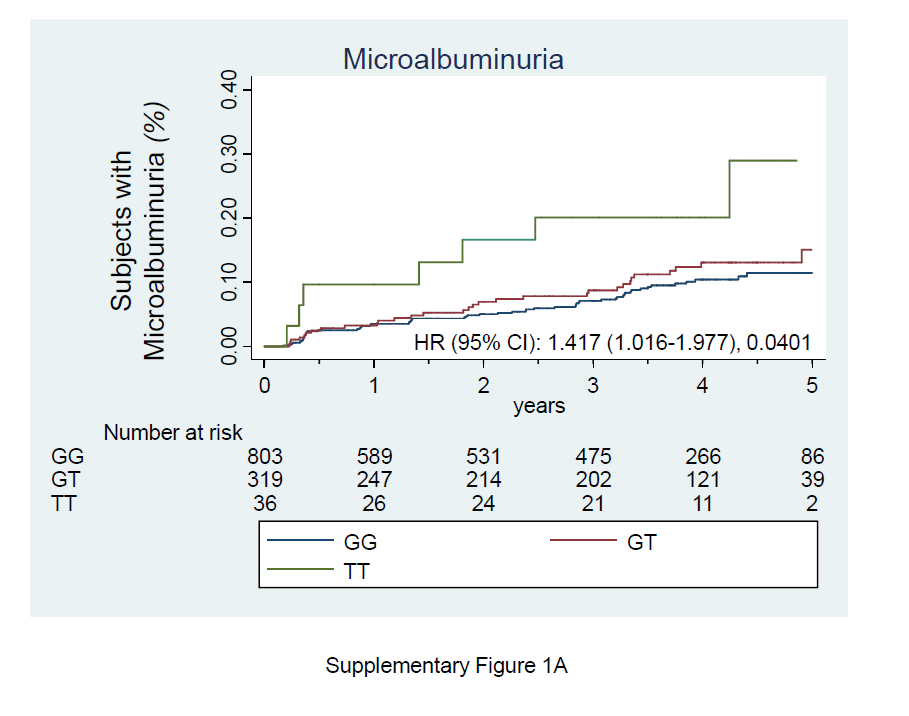


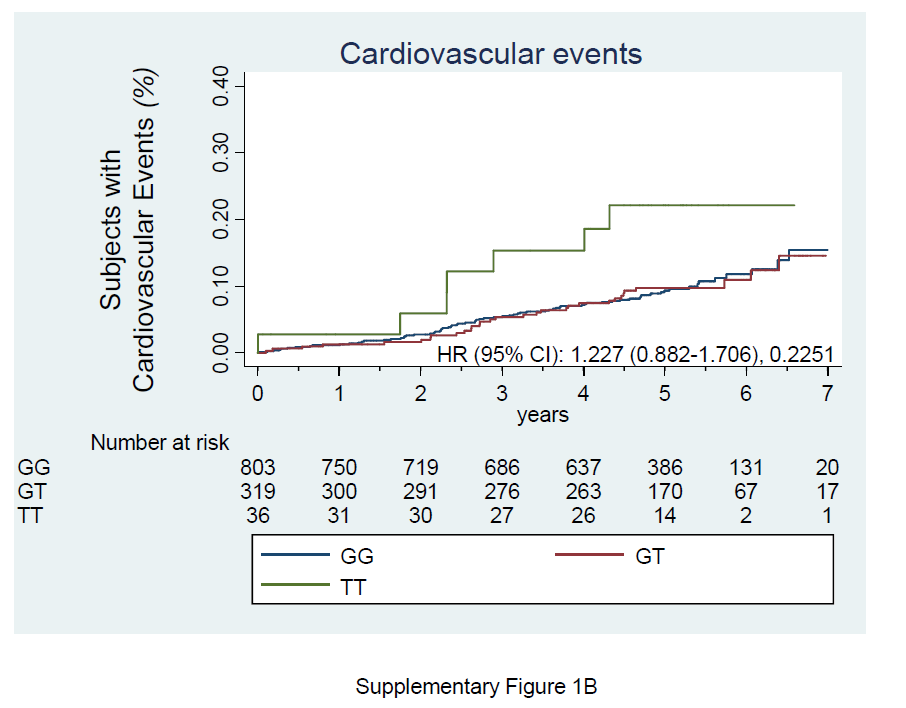

Supplement: Supplementary file 1 [file DataSheet_1.doc]
